# Supplementary figures and images for: Identification of Immunogenic Cell Death-Related Signature for Glioma to Predict Survival and Response to Immunotherapy
Source: Cancers (Basel). 2022 Nov 18;14(22):5665. doi: 10.3390/cancers14225665 (PMC9688866; doi:10.3390/cancers14225665)

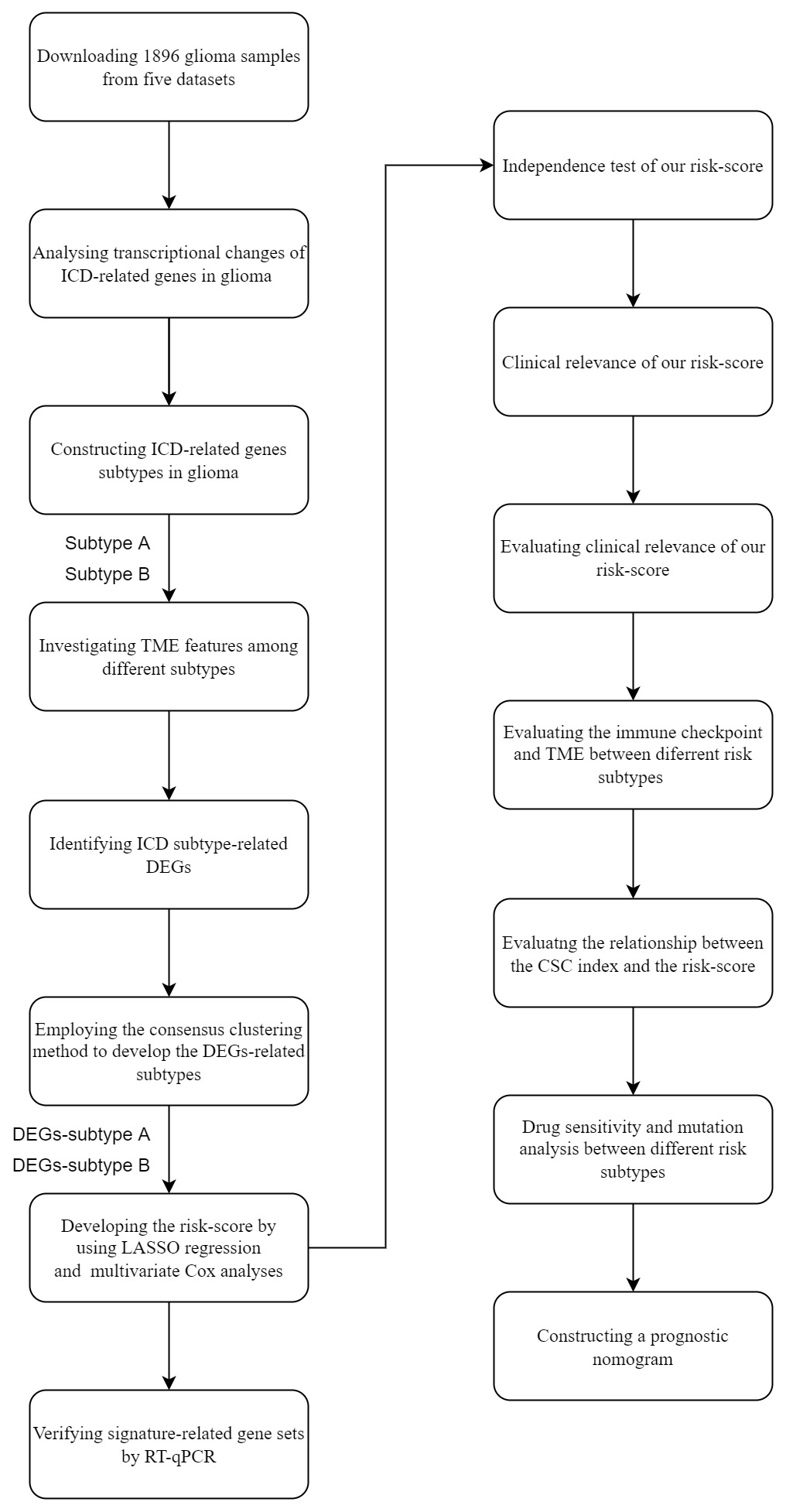

Supplement: Supplementary file 1 [file cancers-14-05665-s001.zip › Supplementary Figure S1.jpg]

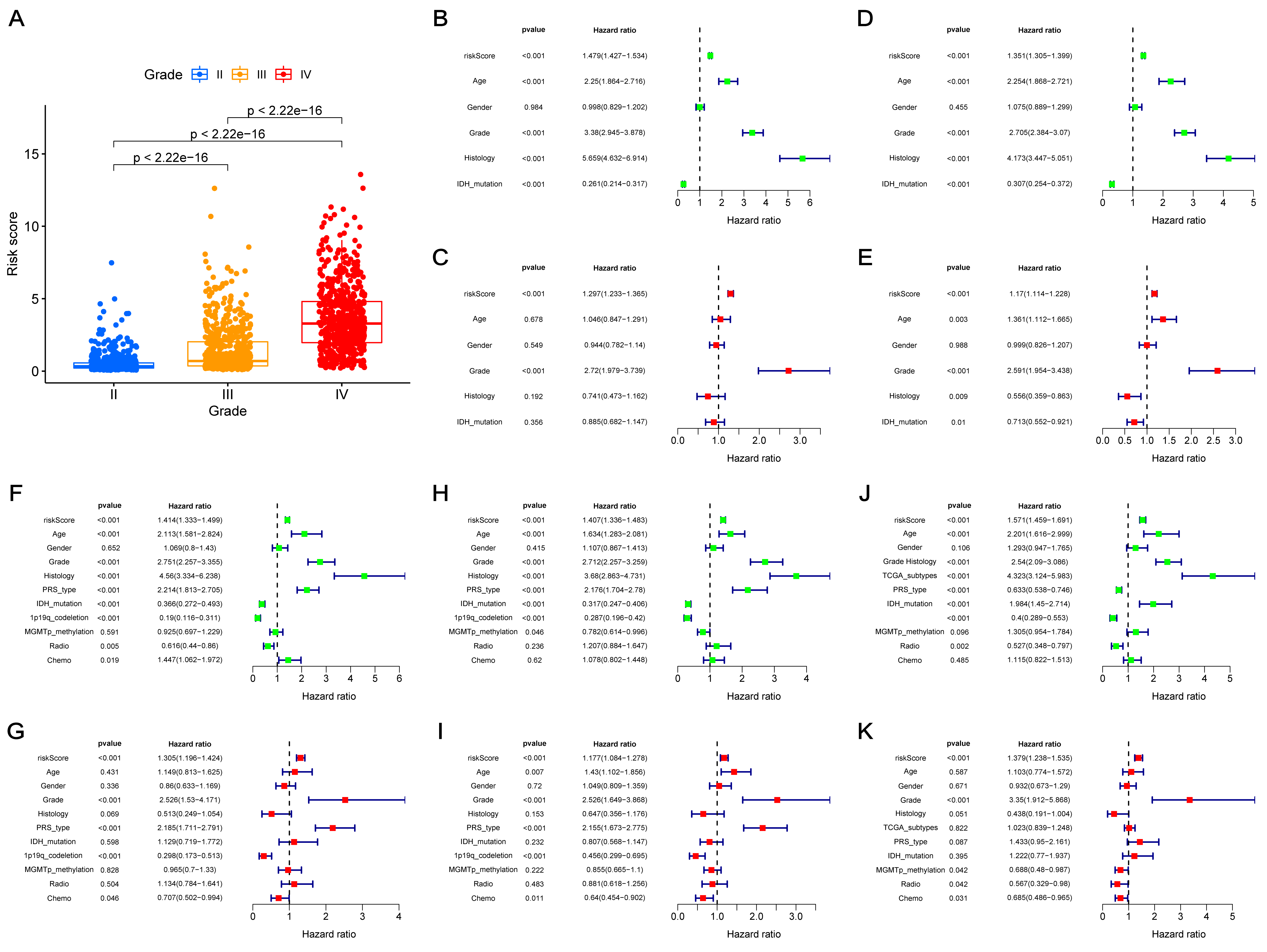

Supplement: Supplementary file 1 [file cancers-14-05665-s001.zip › Supplementary Figure S10.tif]

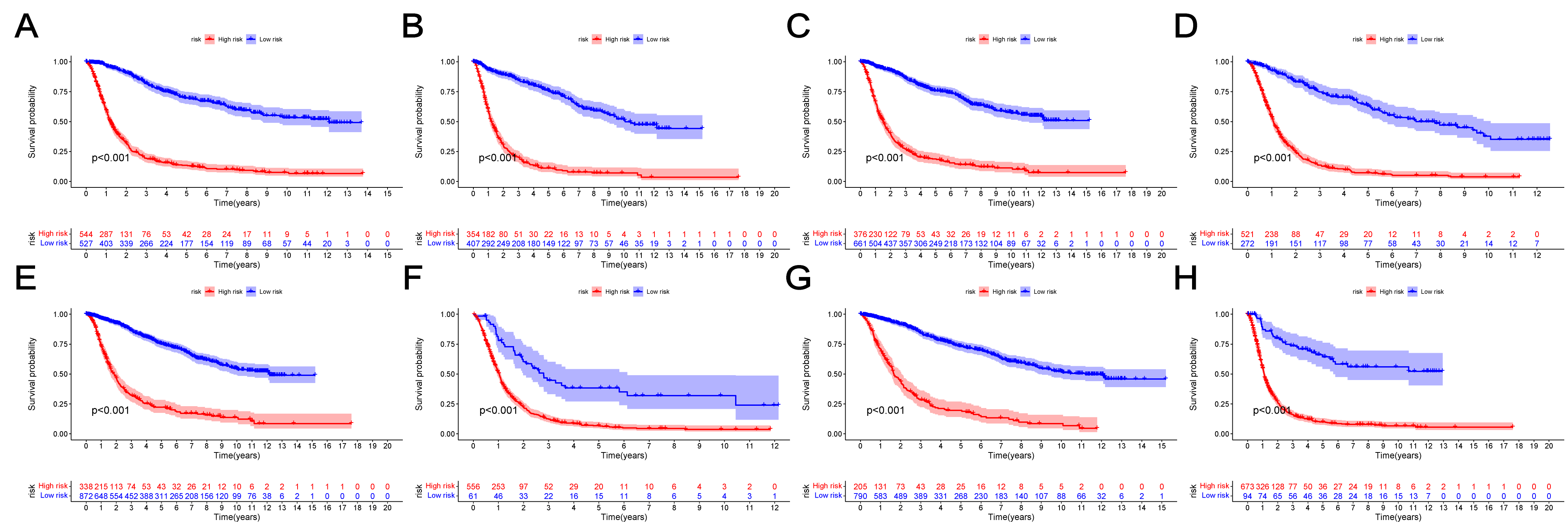

Supplement: Supplementary file 1 [file cancers-14-05665-s001.zip › Supplementary Figure S11.tif]

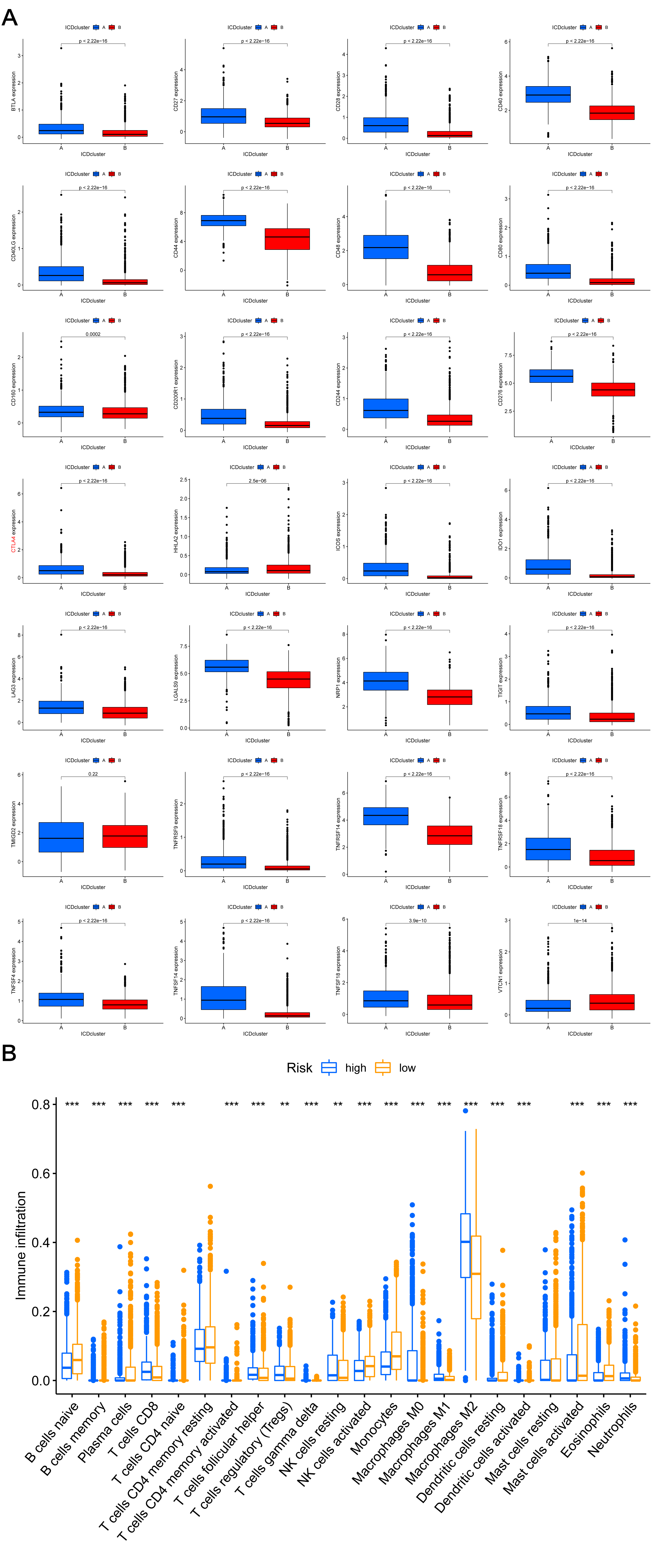

Supplement: Supplementary file 1 [file cancers-14-05665-s001.zip › Supplementary Figure S12.tif]

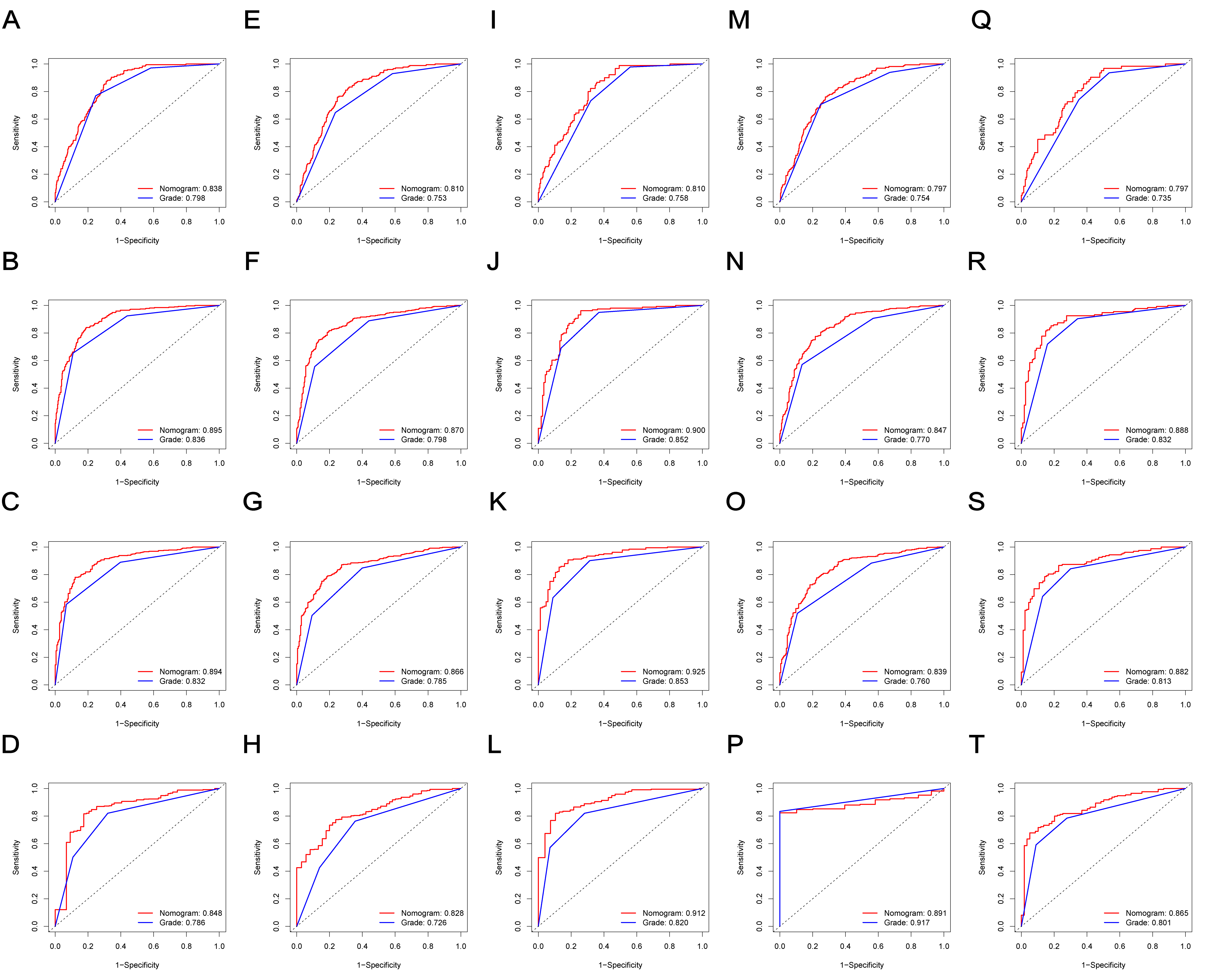

Supplement: Supplementary file 1 [file cancers-14-05665-s001.zip › Supplementary Figure S13.tif]

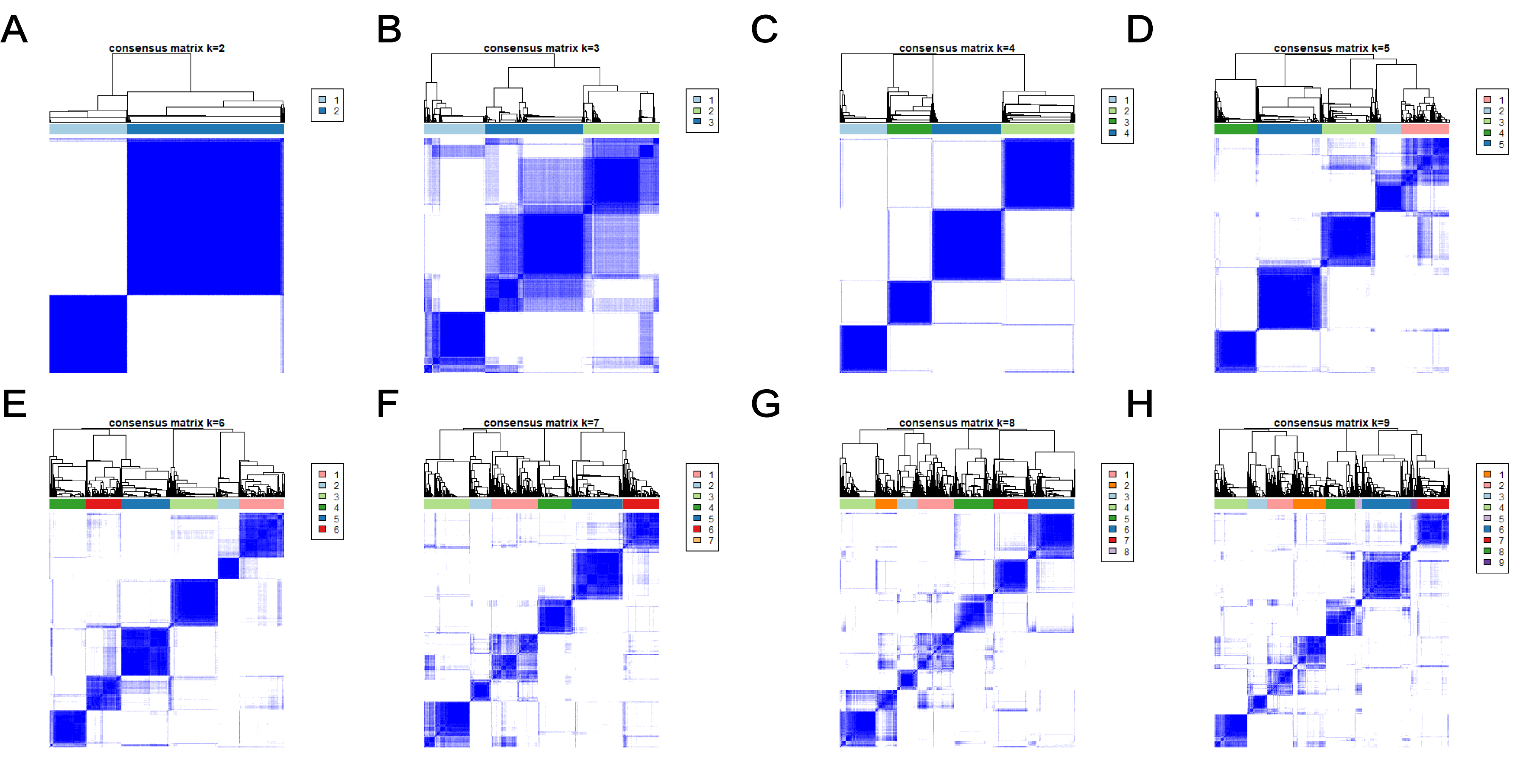

Supplement: Supplementary file 1 [file cancers-14-05665-s001.zip › Supplementary Figure S2.tif]

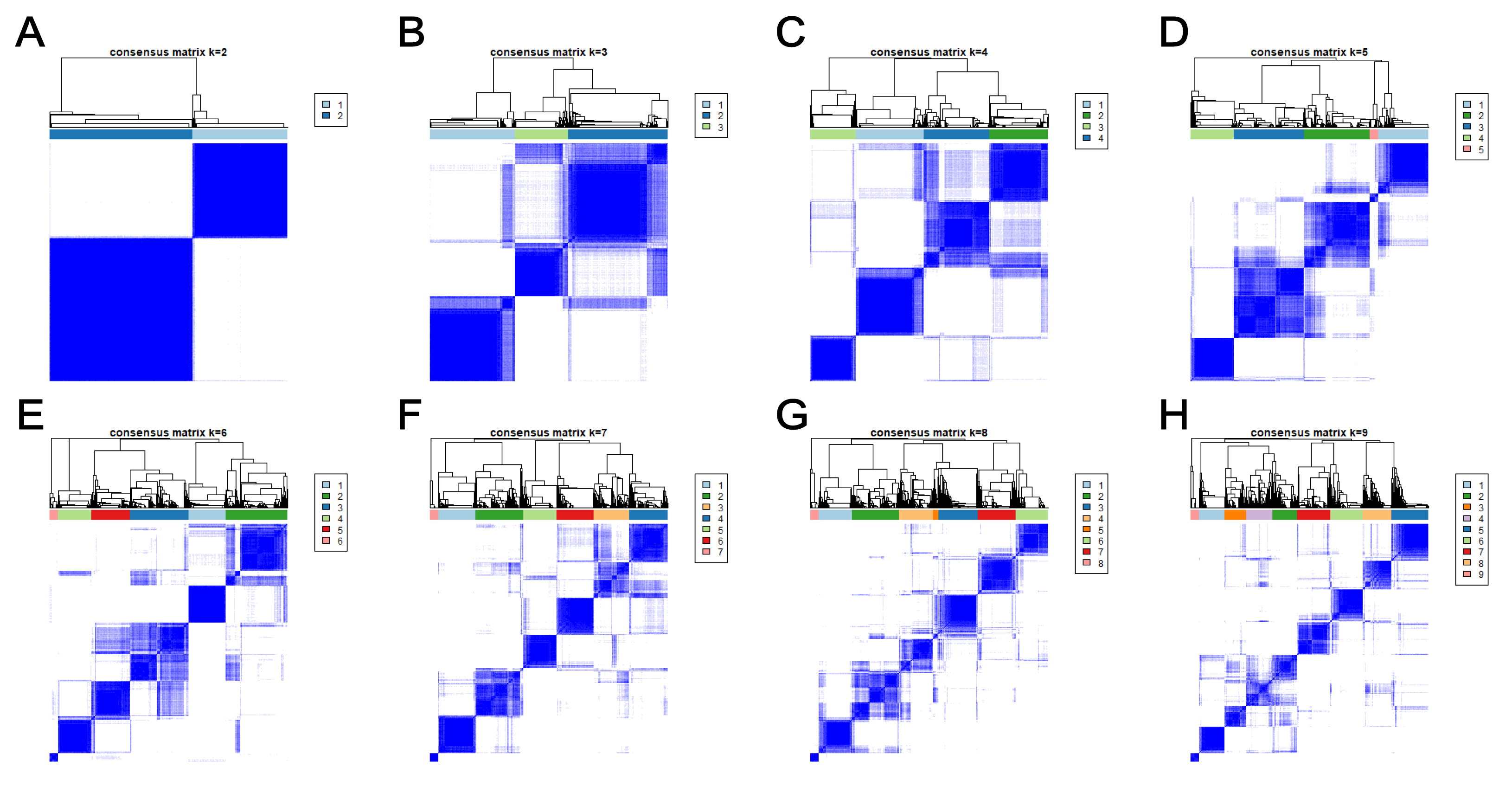

Supplement: Supplementary file 1 [file cancers-14-05665-s001.zip › Supplementary Figure S3.tif]

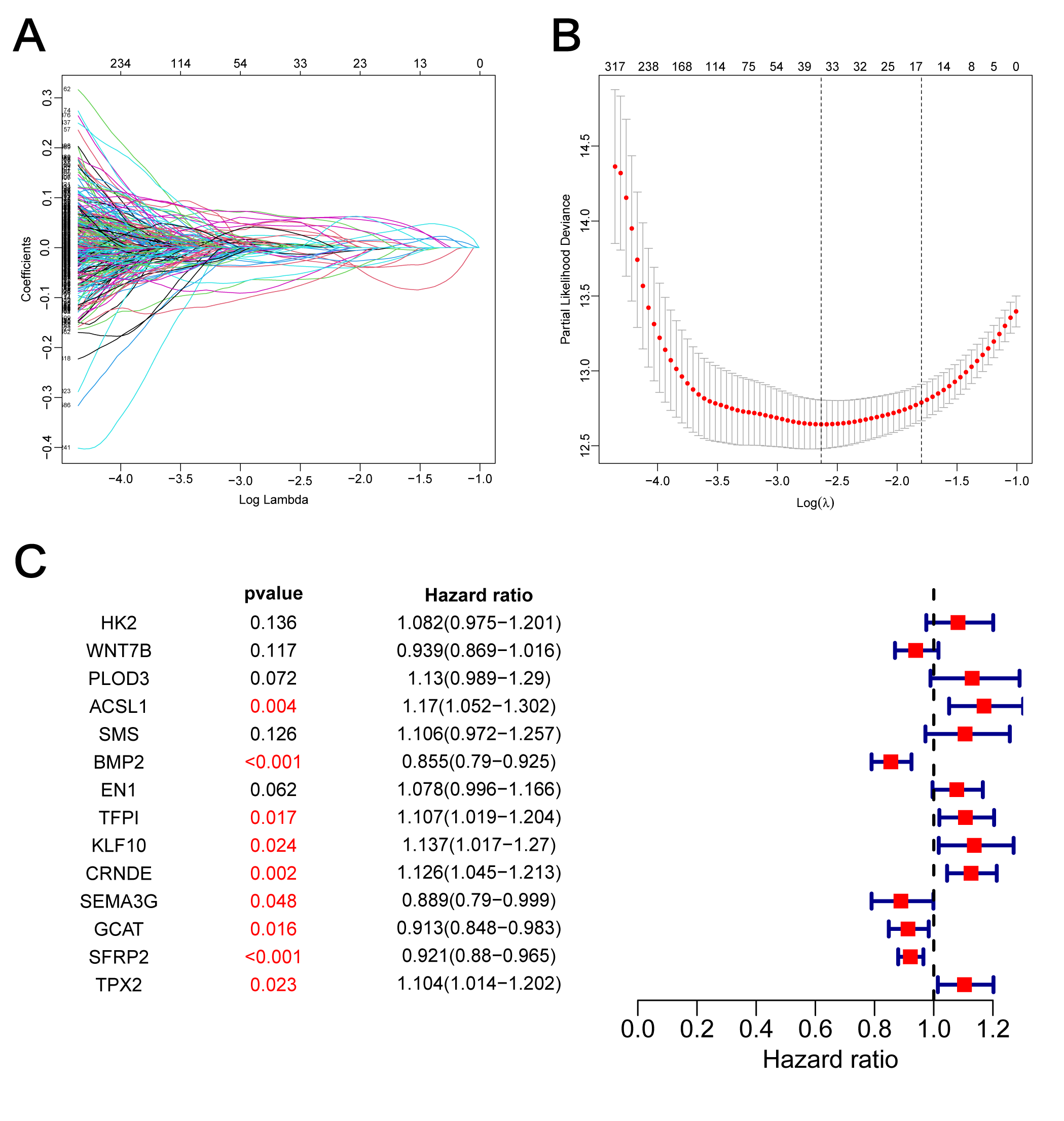

Supplement: Supplementary file 1 [file cancers-14-05665-s001.zip › Supplementary Figure S4.tif]

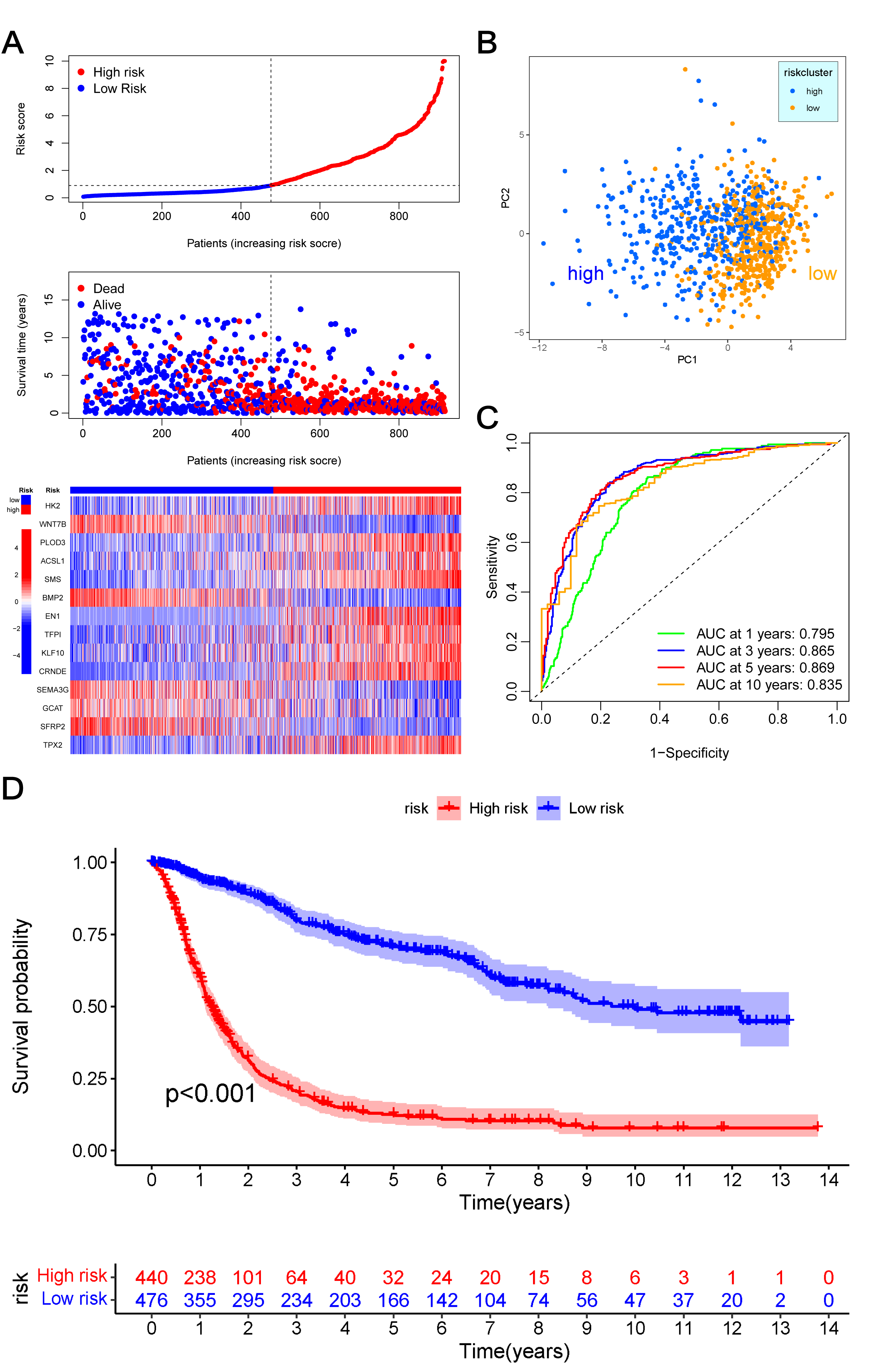

Supplement: Supplementary file 1 [file cancers-14-05665-s001.zip › Supplementary Figure S5.tif]

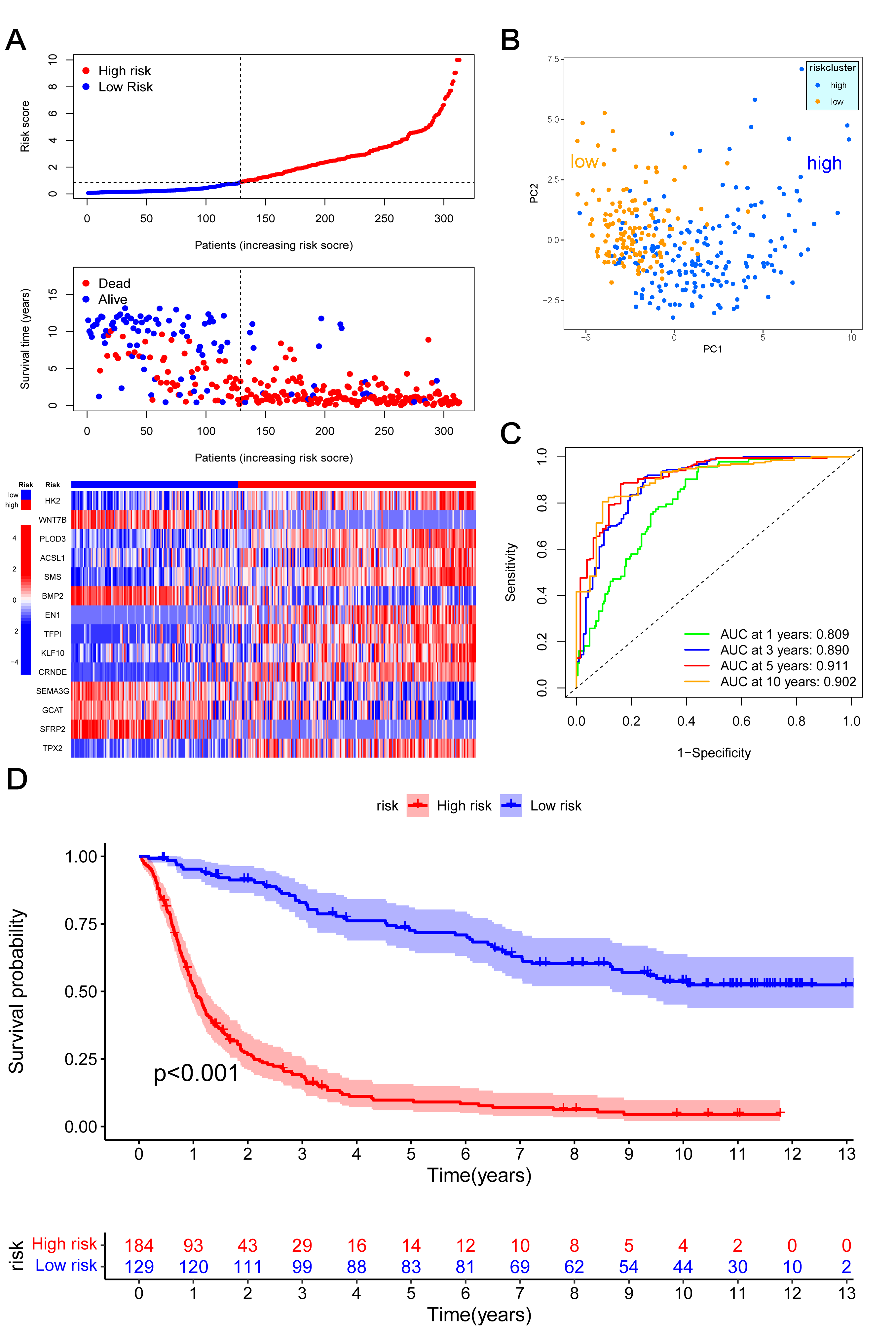

Supplement: Supplementary file 1 [file cancers-14-05665-s001.zip › Supplementary Figure S6.tif]

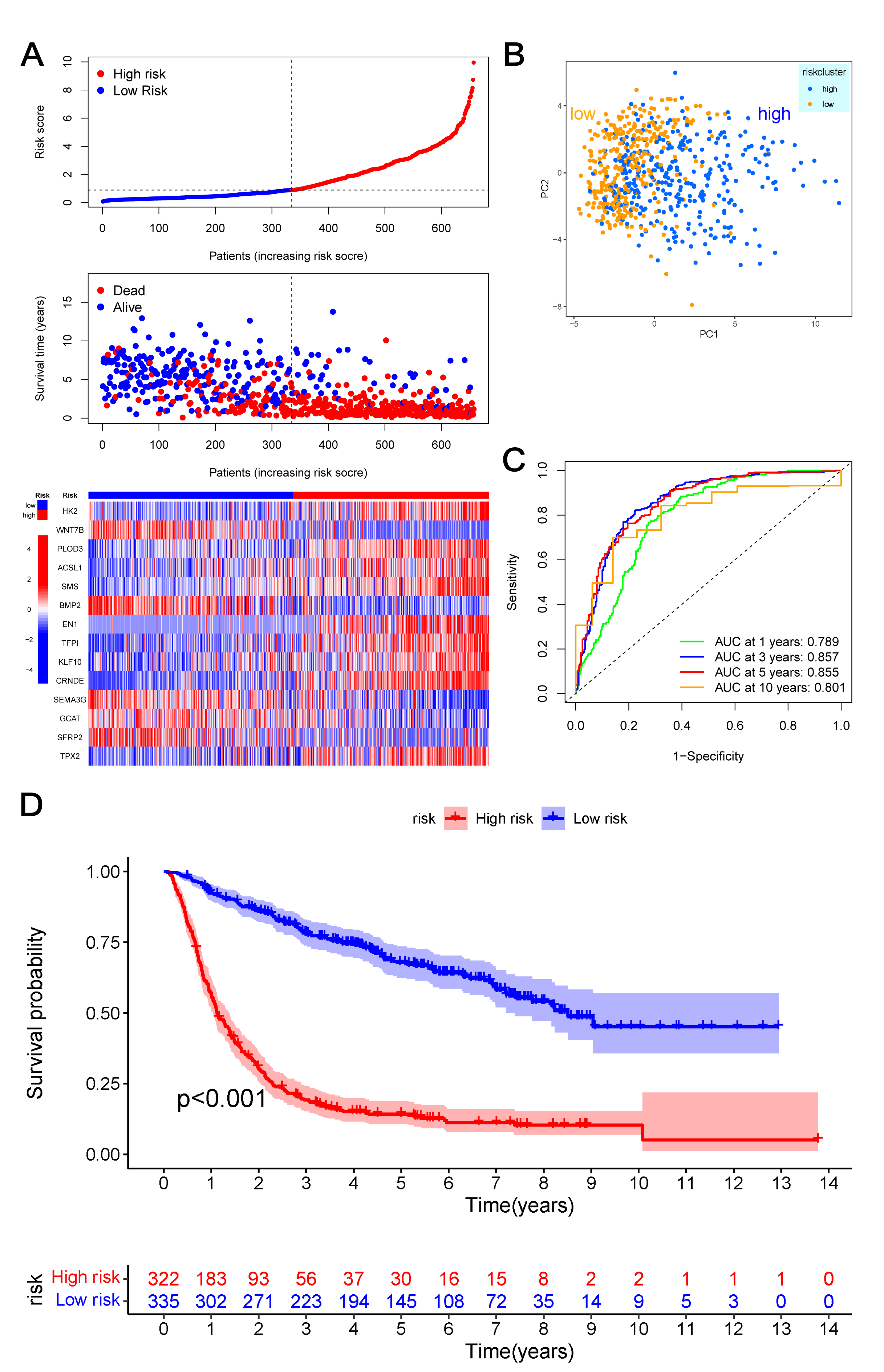

Supplement: Supplementary file 1 [file cancers-14-05665-s001.zip › Supplementary Figure S7.tif]

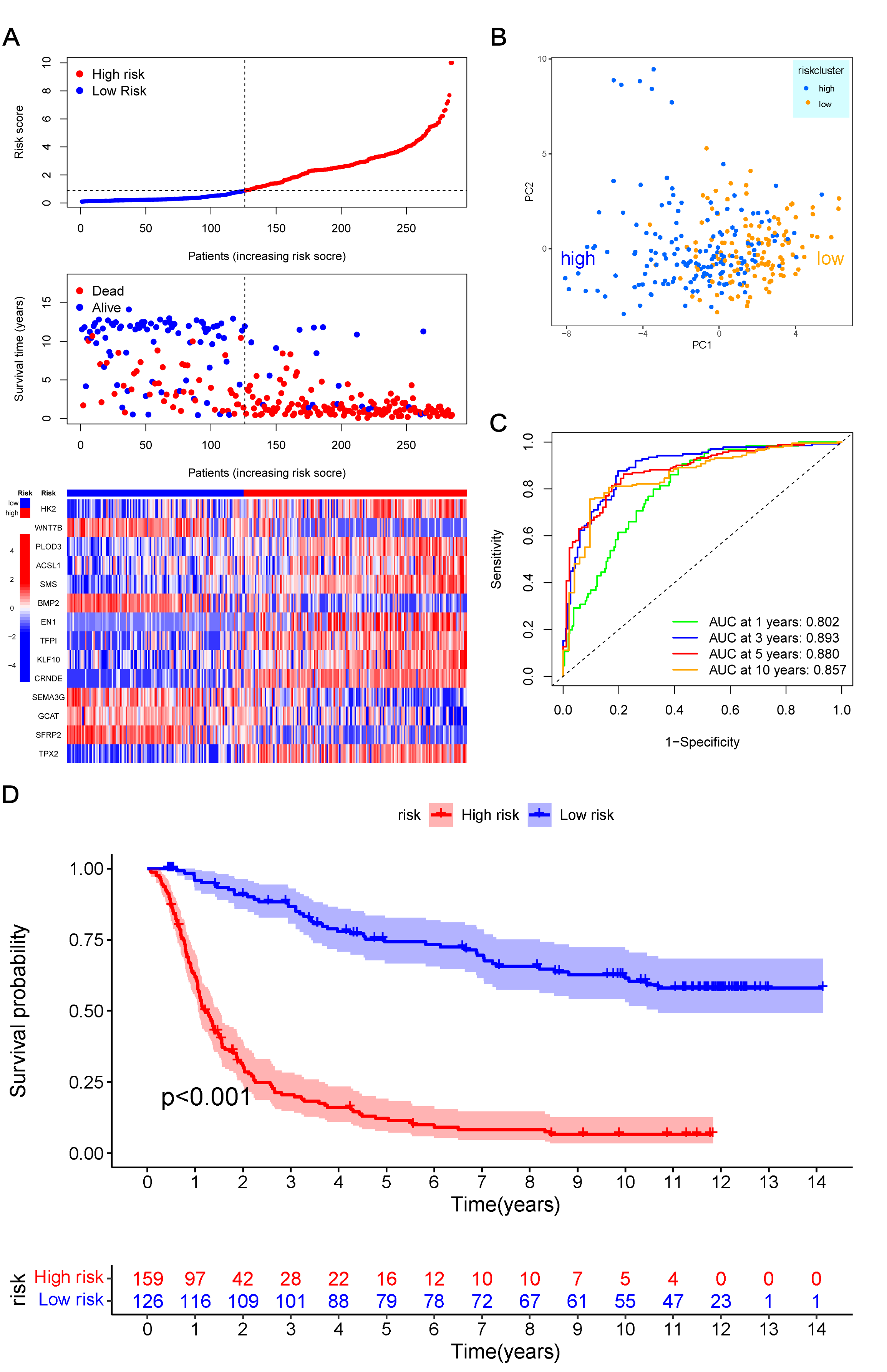

Supplement: Supplementary file 1 [file cancers-14-05665-s001.zip › Supplementary Figure S8.tif]

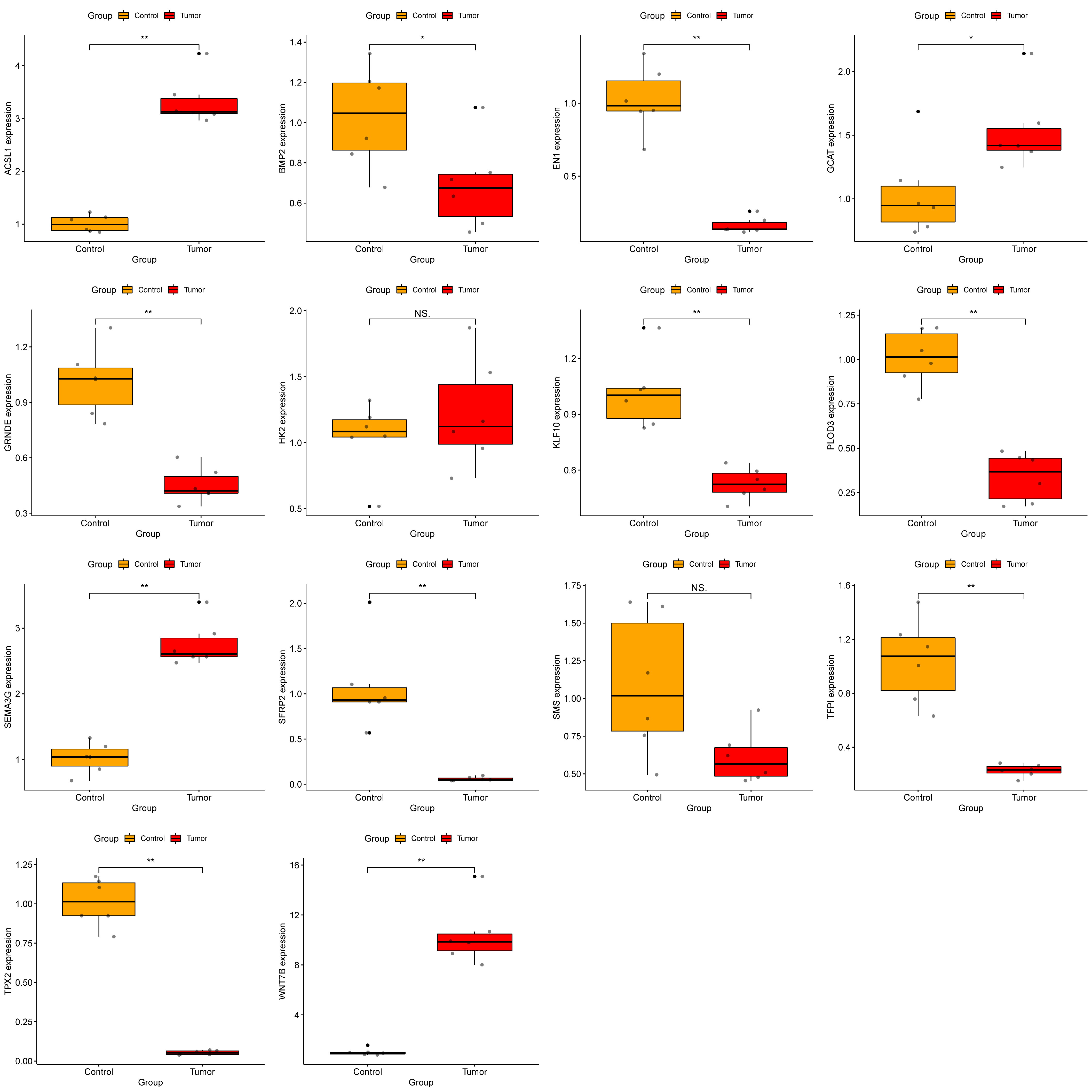

Supplement: Supplementary file 1 [file cancers-14-05665-s001.zip › Supplementary Figure S9.tif]
